# Combined ultrasound and nerve stimulator-guided deep nerve block may decrease the rate of local anesthetics systemic toxicity: a randomized clinical trial

**DOI:** 10.1186/s12871-019-0750-6

**Published:** 2019-06-12

**Authors:** Xu-hao Zhang, Yu-jie Li, Wen-quan He, Chun-yong Yang, Jian-teng Gu, Kai-zhi Lu, Bin Yi

**Affiliations:** Department of Anesthesia, Southwest Hospital, Army Medical University, Chongqing, 400038 China

**Keywords:** Ultrasound, Nerve stimulation, Nerve block, Female, HBV, LAST

## Abstract

**Background:**

Ultrasound guidance might decrease the incidence of local anesthetics systemic toxicity (LAST) for many peripheral nerve blocks compared with nerve stimulator guidance. However, it remains uncertain whether ultrasound guidance is superior to nerve stimulator guidance for deep nerve block of the lower extremity. This study was designed to investigate whether deep nerve block with ultrasound guidance would decrease the incidence of LAST compared with that with nerve stimulator guidance, and to identify associated risk factors of LAST.

**Methods:**

Three hundred patients undergoing elective lower limb surgery and desiring lumbar plexus blocks (LPBs) and sciatic nerve blocks (SNBs) were enrolled in this study. The patients were randomly assigned to receive LPBs and SNBs with ultrasound guidance (group U), nerve stimulator guidance (group N) or dual guidance (group M). The primary outcome was the incidence of LAST. The secondary outcomes were the number of needle redirection, motor and sensory block onset and nerve distribution restoration time, as well as associated risk factors.

**Results:**

There were 18 patients with LAST, including 12 in group U, 4 in group N and 2 in group M. By multiple comparisons among the three groups, we found that the incidence of LAST in group U (12%) was significantly higher than that in group N (4%)(*P* = 0.037) and group M(2%)(*P* = 0.006). The OR of LAST with hepatitis B (HBV) infection and the female sex was 3.352 (95% CI,1.233–9.108, *P* = 0.013) and 9.488 (95% CI,2.142–42.093, *P* = 0.0004), respectively.

**Conclusions:**

Ultrasound guidance, HBV infection and the female sex were risk factors of LAST with LPBs and SNBs. For patients infected with HBV or female patients receiving LPBs and SNBs, we recommended that combined ultrasound and nerve stimulator guidance should be used to improve the safety.

**Trial registration:**

This study was approved by the Ethical Committee of the First Affiliated Hospital of Army Medical University. The protocol was registered prospectively with the Chinese Clinical Trial Registry (ChiCTR-IOR-16008099) on March 15, 2016.

## Background

Although peripheral nerve blocks have been a safe and effective way to provide analgesia for procedures in a variety of settings, using this type of anesthesia does have risks that should not be overlooked. The incidence of Local Anesthetics Systemic Toxicity (LAST) was reported to be 0.04/1000 to 1.8/1000 in a recent summary [1]. LAST, a life-threatening and sometimes fatal condition, was reported to be related to patient characteristics (such as advanced age, low muscle mass, liver disease, cardiac disease, renal disease or diabetes), local anesthetic characteristics and practice settings [1].

Lumbar plexus blocks (LPBs) combined with sciatic nerve blocks (SNBs) for lower extremity surgery are becoming increasingly popular. Due to the depth of the lumbar plexus and sciatic nerves, LPBs and SNBs were advanced regional anesthesia techniques and may be more likely to lead to LAST [2]. LPBs and SNBs are traditionally performed using surface anatomical landmarks and nerve stimulation guidance. Ultrasound could offer direct visualization of the nerve structures, needle pathway and local anesthetics (LAs) spread in real time and is thus widely used in peripheral nerve blocks. Accumulating published data suggests higher efficacy and safety of nerve blocks with ultrasound guidance (US) [3, 4], specifically for interscalene [5], supraclavicular [6], infraclavicular [7], and axillary [8] blocks. Michael et al. [9] reported that the use of ultrasound reduced the risk of LAST throughout its continuum by 60 to 65% compared to without ultrasound. However, most studies have focused on upper extremity blocks. Due to the deep location of the lumbar plexus and sciatic nerves, whether the use of ultrasound in LPBs and SNBs would be beneficial in terms of efficacy and safety remains a matter of debate. Most relevant published studies have suggested that ultrasound guidance would shorten both the time required to perform the block and the onset time [10–12]. However, there are limited studies comparing the incidence of LAST for LPBs and SNBs with ultrasound and nerve stimulator.

We designed this study to determine whether ultrasound guidance deep nerve blocks would decrease the incidence of LAST compare with nerve stimulation guidance and to identify associated risk factors of LAST.

## Methods

This study was approved by the Ethical Committee of the First Affiliated Hospital of Army Medical University (previous name: Southwest Hospital of the Third Military Medical University). The protocol was registered prospectively with the Chinese Clinical Trial Registry (ChiCTR-IOR-16008099) on March 15, 2016. The principal investigator was Bin Yi. The study took place at the Department of Anesthesia, the First Affiliated Hospital, Army Medical University, Chongqing, from August 25, 2016, to August 14, 2017.

Patients scheduled for elective lower limb surgery in the Southwest Hospital and desiring LPBs and SNBs were offered enrolment. Written informed consent was obtained from the participants for publication of this article and any accompanying Tables. A copy of the written consent is available for review by the Editor of this journal. The inclusion criteria were as follows: willingness to participate in the study (written informed consent); ASA classification of I to III; older than 18 years old. The exclusion criteria were as follows: refusal to participate; history of neurological diseases; coagulopathy or infection at the site of the block, allergy to local anesthetics (LAs), and any contraindication to peripheral nerve blockade noted by the attending anesthesiologist. All patients were randomly allocated to group U (ultrasound guidance), group N (nerve stimulator guidance) or group M (combined guidance) by a random number table.

## Blinding

The anesthesiologist who performed the LPBs and SNBs was strictly blinded to the patient’s group assignment before the procedure. Only when the anesthesiologist commenced with the block was a prepared sealed opaque envelope containing the patient’s group assignment opened. Then the anesthesiologist completed the block with the indicated technique. There were two investigators in the study. One investigator blinded to the technique used was present in the block area to assess the procedure-related outcomes. To ensure blindness of the patient to the method, all procedures performed behind an opaque screen and investigators were required not to say anything about the technique in use. Another investigator assessing the block quality was blinded to the group allocation and remained outside the block area until completion of the procedure. Finally, a statistician blinded to the entire process performed the statistical analysis, with group data labelled only as numbers until all analyses were completed.

## Block preparation

LPBs and SNBs were performed preoperatively by 1 of 3 attending anesthesiologists who were skilled in peripheral nerve blocks with both ultrasound guidance (US) and nerve stimulator guidance (NS). All of them had been in clinical practice with a focus on regional anesthesia for at least 5 years. After arriving in the operating room, the patient was placed in the lateral decubitus position with the surgical limb uppermost and monitored continuously via electrocardiography, SpO_2_ measurements, and non-invasive blood pressure monitoring during the nerve blockade and surgery. Both the ultrasound and nerve stimulation systems were prepared and positioned conventionally in each group. The ultrasound machine and nerve stimulator were turned on, and a grounding lead was placed on the lateral aspect of the leg being blocked for each group. The patient’s group allocation was given to the anesthesiologist only after the preparation of both systems and just before the block procedure. The patient was pretreated with 0.05 mg/kg midazolam and 1.5 μg/kg fentanyl. The injection site was prepared with chlorhexidine gluconate. Five millilitres of 0.5% lidocaine was injected subcutaneously at the site of needle insertion. The LA mixture was composed of 200 mg of ropivacaine, 200 mg of lidocaine and 20 ml of 0.9% sodium chloride solution. The concentration of ropivacaine and lidocaine was 0.4%. The total amount of LAs used was determined by the dosage of ropivacaine needed, namely, 3 mg/kg. The patient and investigators assessing the block quality prevented from seeing both the block procedure itself and the sonographic image displayed by an opaque screen. According to the group allocation, the patient received the nerve blocks under one of the following three techniques.

### Nerve stimulation technique

In the operating room, LPB was performed using Chayen’s approach [13, 14]. The puncture site was located 4–5 cm lateral to the posterior midline along the intercristal line. A 110-mm, 22-G stimulating needle connected to a nerve stimulator (Stimuplex HNS 11, B. Braun) was advanced perpendicular to the skin. The nerve stimulator was set to a pulse duration of 0.1 ms, current intensity of 1.0 mA, and frequency of 2 Hz. The stimulating intensity was progressively reduced to 0.4 mA or less while maintaining the twitch in the quadriceps distribution. The total volume of LAs was determined by the amount of the LA mixture calculated according to the patient’s weight, as mentioned above. Each point was given half of the total calculated volume of the LA mixture. When the correct needle position was achieved based on evoking the desired motor response, the amounts of LAs described above were injected. SNB was performed with the classic Labat approach [15]. The needle was inserted 5 cm below the midpoint of a line connecting the posterior superior iliac spine and the greater trochanter. After an appropriate stimulus was localized in the sciatic distribution, the LAs described above were injected.

### Ultrasound-guided technique

We chose the “Shamrock Method” for the LPBs [16]. A sterile cover was placed on a 3-MHz low-frequency ultrasound probe (LOGIQe 4C-RS, GE Inc., USA). The ultrasound transducer was positioned on the line connecting the subcostal margin and iliac spine and adjusted until a clear view of the psoas, erector muscle and quadratus lumborum appeared. The hyperechoic structure located in the posterior internal quadrant of the psoas was the lumbar plexus. The puncture site was beneath the probe and 4–5 cm lateral to the vertebral body. We chose a subgluteal approach for the SNBs [17]. The ultrasound transducer was positioned perpendicular to the skin on the line connecting the ischial tuberosity and greater trochanter, and a clear transverse image of the hyperechoic sciatic nerve between the ischial tuberosity and greater trochanter was obtained. For the LPBs and SNBs, the needle placement and LA spread were confirmed by ultrasound visualization. After the proper needle placement was confirmed, incremental injection of the same LA solution in the same volume was performed as previously described until circumferential spread around the nerve was obtained. The needle was redirected, when required, to achieve this goal. In group M, initially, needle-to-nerve guidance was applied as in group U. Maintaining the needle nerve position, the nerve stimulator was set as described for group N. When the correct needle position was achieved based on evoking the desired motor response, the LAs described above were injected.

### Block evaluation

Evaluation of the nerve blocks was performed by an investigator blinded to those who administered the LPBs and SNBs. The motor and sensory responses in the nerve distribution area were assessed every 5 min until complete motor and sensory effects were achieved. If it took more than 30 min to achieve sensory loss in both distributions after the end of the LAs injection, the block was considered to have failed. The attending anesthesiologist had the right to perform general anesthesia, rescue block, or supplementation with a local field block in case of a failed block. The motor block was assessed with a modified Bromage scale: 2, full motor strength; 1, decreased strength; and 0, no strength. Similarly, the sensory block was evaluated with ice: 2, full sensation (no change); 1, decreased sensation; and 0, no sensation.

Postoperative follow-ups were performed in the post-anesthesia care unit and by telephone within 72 h after the procedure by clinical personnel in addition to study-related procedures.

### Outcomes

The primary outcome was the incidence of LAST. LAST can present with clinical manifestations related to both the central nervous system (CNS) and the cardiovascular system (CVS). CNS symptoms include tongue numbness, tinnitus, light-headedness, metallic taste, nystagmus, confusion, tremors, agitation, seizures, coma, and respiratory arrest [18]. CVS symptoms include tachycardia, arrhythmias, hypertension, and later toxic symptoms, such as bradycardia, cardiac depression, cardiovascular collapse, and asystole [18]. The secondary outcomes were the quality of the nerve block and associated risk factors. The quality of the block included the number of needle redirections, motor and sensory block onset and restoration times in the lumbar and sciatic nerve distributions. The associated risk factors included age, sex and comorbidities. The number of needle redirections was counted as the number of times the needle was withdrawn by at least 10 mm with subsequent forward movement. The upper limit of redirections was 20, but if necessary, the needle was allowed to be redirected as many times as possible to achieve proper placement, as previously described. The onset of motor and sensory block was assessed using the modified Bromage scale as mentioned above for the distributions of both the lumbar plexus and sciatic nerves. The onset time was measured between the final LA injection and the first observation of a 0 score. During the phone follow-up interviews, the patient provided the time of first return of sensation and any block-related complications on postoperative day 1. The block duration time was defined as the interval between block completion and the first return of sensation. Any reported complications were recorded.

### Statistical analysis

The statistical analyses were performed using Statistical Package for the Social Sciences (Windows Software, version 19.0; SPSS Inc., Chicago, IL) and Power Analysis and Sample Size (Windows Software, version 11.0; NCSS Inc., Utah).

Demographic and perioperative data are expressed as the mean and standard deviation. Parametric and non-parametric Kolmogorov–Smirnov tests were applied to assess normality. The primary outcome (incidence of LAST) and potential risk factors were compared by χ^2^ test or Fisher exact test when appropriate (*n* < 5 in any field). In the χ^2^ test, we tested whether there were differences in the incidence of LAST and the odds ratios of potential risk factors among different groups. The demographics and secondary outcomes were compared among the three groups by one-way ANOVA, followed by multiple comparisons using the LSD test or Welch and Dunnett’s T3 test for unequal variances. Using one-way ANOVA, we tested whether there were differences in the patient characteristics and block quality among the three groups. This analysis was followed by the determination of 95% CIs with Bonferroni’s correction to adjust for multiple comparisons (three different methods for nerve block for motor and sensory onset, restoration time and demographics) to minimize the chance of a type I error (0.05). For all comparisons, 2-tailed *P* values < 0.05 were considered statistically significant.

The incidence of LAST is low according to published data. Therefore, we performed a test to determine the power of the analysis regarding the incidence of LAST in the three groups after the experiment. In the current study, 319 patients were randomly allocated to the three groups. Finally, data from 100 patients for each group were analysed. We performed a test to determine the power of the analysis regarding the primary outcome after the experiment. We calculated the effect size (0.182) using PASS software. Then, we set the significance level to 0.05. We found that when the total sample size was 300, the power(1-β) of the test was 0.81.

## Results

**Fig. 1:**
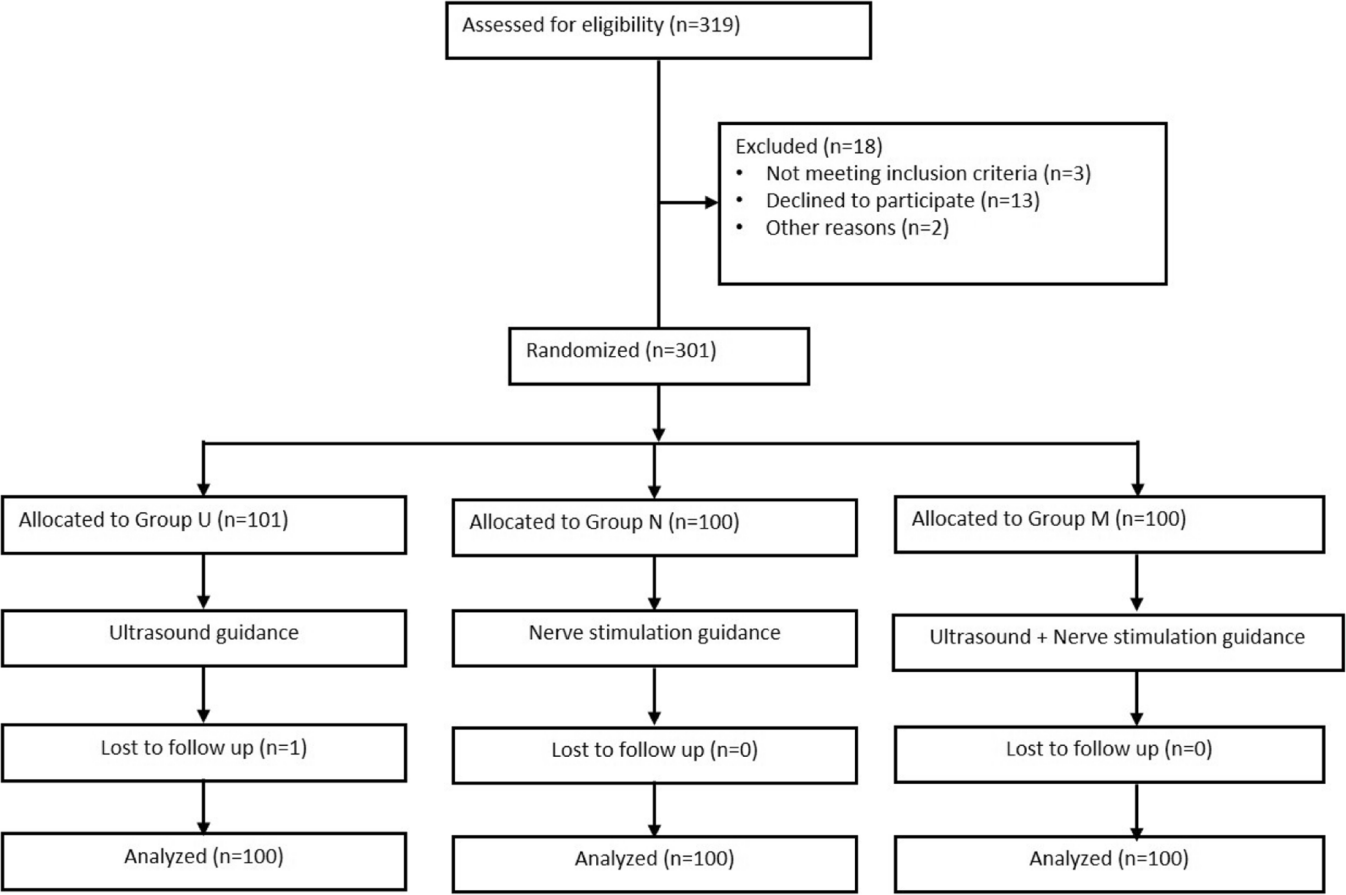
CONSORT flow diagram of the study. CONSORT indicates Consolidated Standards of Reporting Trials. Group U was short for nerve block with ultrasound guidance, group N was short for nerve block with nerve stimulation guidance, group M was for nerve block with combined guidance

The study flow diagram is presented in Fig. 1. A total of 319 patients were evaluated for eligibility and offered enrolment in this study. Eighteen were excluded; 3 did not meet the inclusion criteria, 13 declined to participate, and 2 were excluded for other reasons. There were no failed or aborted blocks in either group. One patient in group U was lost to follow-up.

The patient characteristics are presented in Tab 1. There was no statistically significant difference in age, sex, weight or height among the three groups. Only one patient in group M had an ASA III status. Most of the operations were performed on the knee or ankle. There was a statistically significant difference in the operative time between group U (41.0 ± 24.21 min) and in group M (51.5 ± 30.8 min).

**Table 1:** Patients characteristics

	group U(100)	group N(100)	group M(100)	*F*	*P*
Age (yr) (SD)	41.7(12.85)	39.9 (14.71)	42.0 (19.94)	0.6222	0.53745
Gender (F/M)	55/45	47/53	43/57		(N vs U)0.51/(M vs U)0.49
Weight (kg) (SD)	63.8 (11.92)	64.7(11.06)	64.7(11.20)	0.2185	0.8038
Height (cm) (SD)	163.0 (8.07)	165.0 (9.68)	164.9 (9.3)	1.5282	0.2186
Surgical duration (min)	41.0(24.21)	46.9 (32.26)	51.5(30.8)	3.1996	0.04219
Surgical site(knee/ankle/other)	89/10/1	78/21/1	84/16/0		
ASA I/II/III	53/47/0	48/52/0	49/50/1		

Data are expressed as mean ± SD

*Abbreviations*: *group U* nerve block with ultrasound guidance, *group N* nerve block with nerve stimulation-guidance, *group M* nerve block with combined guidance, *F* female, *M* male, *ASA* American Society of Anesthesiologists

The primary and secondary outcomes are shown in Tab 2. The incidence of LAST in all three groups was 6%. Moreover, there was a statistically significant difference in the incidence of LAST among the three groups. By multiple comparisons among the three groups, we found that the incidence of LAST in group U (12%) was significantly higher than that in group N (4%)(*P* = 0.037) and group M (2%)(*P* = 0.006). (shown in Tab 4). Regarding the LPBs, the motor onset time was significantly shorter in group N (9.5 ± 3.55 s) than in group U (11.30 ± 4.94 s) and group M (11.10 ± 4.38 s) (shown in Tab 2). There was no statistically significant difference in the sensory onset time or sensory and motor restoration time among the three groups. Regarding the SNBs, the motor and sensory onset time was significantly shorter in group N than in groups U and M. Meanwhile, the sensory and motor restoration time in group N was statistically significantly longer than that in groups U and M.

**Table 2:** Outcomes

Outcomes	group U(100)	group N(100)	group M(100)	*P*
Incidence of LAST(%)	4%	12%	2%	0.007
Motor onset, Lumbar plexus, min (SD)	11.3(4.94)	9.5 (3.55)	11.1 (4.38)	0.00729
Motor onset, Sciatica, min (SD)	15.1 (4.04)	13.4 (3.03)	15.0 (3.20)	0.00041
Sensory onset, Lumbar plexus, min (SD)	8.5(3.64)	7.8 (2.52)	8.8 (2.88)	0.07676
Sensory onset, Sciatica, min (SD)	9.6 (2.62)	8.8 (1.59)	9.5 (1.79)	0.02399
Sensory restoration, Lumbar plexus, h (SD)	8.0 (1.90)	8.4 (1.71)	8.1 (1.63)	0.19313
Sensory restoration, Sciatica, h (SD)	7.1 (1.73)	7.7 (1.63)	7.0 (1.57)	0.00329
Motor restoration, Lumbar plexus, h (SD)	8.9 (2.11)	9.3 (1.78)	9.0 (1.59)	0.18912
Motor restoration, Sciatica, h (SD)	7.9(1.87)	8.5 (1.72)	7.8 (1.44)	0.00760

Data are expressed as mean ± SD or number with %

*Abbreviations*: *group U* nerve block with ultrasound guidance, *group N* nerve block with nerve stimulation-guidance, *group M* nerve block with combined guidance

Detailed information of the 18 patients who developed LAST is summarized in Tab 3. There were 12 patients from group U (66.7%), 4 from group N (22.2%) and 2 from group M (11.1%) who experienced LAST during the process. Most of the symptoms were CNS symptoms. None of the 18 patients developed permanent complications after correct and timely treatment. To our interest, 16 of the 18 patients were female. The age of the 18 patients ranged from 19 to 81. The shortest occurrence time was 1 min after finishing the block, and the longest occurrence time was 22 min. The shortest duration time was 3 min without any treatment. The longest duration time was 100 min due to the use of propofol.

**Table 3:** Summary of Events of Local Anesthetic Systemic Toxicity (LAST)

Group	Sex	Age(Y)	Weight(kg)	Height(cm)	Signs and Symptoms	Occurrence time	Treatment	Duration time (min)
N	F	35	52.5	155	Lips numbness, Left hand twitch	17	M2	6
N	F	46	55	155	Lips numbness	11	/	5
N	F	65	62.5	163	Agitation, Chest tightness	9	/	3
N	F	19	35.5	149	Tachycardia, Seizures	12/22	M2 P200V	80^a^
U	F	46	54	158	Tongue numbness, Tinnitus	8	M2	9
U	F	53	54	160	Tongue numbness, Left hand and leg twitch	13/17	M2	8
U	F	26	70	161	Tongue numbness	18	/	4
U	F	41	50	156	Unconsciousness, Tachycardia, Hypertension	8	M2 P200	63^a^
U	F	61	60	155	Agitation	9	/	3
U	F	46	60	159	Scream, Unconsciousness	1	M2 P200	100^a^
U	F	32	51	160	Tachycardia	7	M2 D0.5	25
U	M	81	63	152	Right hand twitch, Unconsciousness	4/9	M2	15
U	F	62	58	150	Transient numbness of right hand and leg	20	/	4
U	F	26	46	161	Twitch	12	M2	5
U	F	47	60	145	Hypertension, Tachycardia, Agitation	10	M2	25
U	F	37	48	153	anxiety, Confusion	11	M2	15
M	F	35	64	163	Tinnitus, Whole body numbness	8	M2	10
M	M	21	60	170	Tongue numbness	11	/	6

Treatment: M2 means venous injection of Midazolam 2 mg; P200 means continuous intravenous infusion of Propofol 200 mg with the rate of 3 mg^.^kg^-1.^h^−1^; D0.5 means continuous intravenous infusion of Dexmedetomidine with the rate of 0.5 μg^.^kg^-1.^h^−1^; V means mechanical ventilation. Dosage of local anesthtic: 0.4%Ropivacaine+ 0.4% Lidocaine, 3 mg/kg);

^a^The main reason of long duration time was the use of propofol

We analysed risk factors such as age, sex, liver disease, and diabetes according to *The Third American Society of Regional Anesthesia and Pain Medicine Practice Advisory on Local Anesthetic Systemic Toxicity* [1]. In the current study, 52 patients were infected with HBV, and 7 of these patients experienced LAST. As shown in Tab 4, the OR of LAST for HBV infection and the female sex was 3.352(95% CI,1.233–9.108, *P* = 0.013) and 9.488(95% CI,2.142–42.093, *P* = 0.0004), respectively. However, age, needle passes, renal disease and diabetes did not increase the risk of LAST in the current study. Overall, the use of ultrasound, HBV infection and the female sex may be related to the increased incidence of LAST in the current study.

**Table 4:** Associated risk factors for local anesthetic systemic toxicity

Categorical Variables		No. LAST Events(%)	*OR*	95% CI	*P*
Method of Block	N	4(4)			0.037(N vs U)
	U	12(12)			0.006 (U vs M)
	M	2(2)			0.407 (M vs N)
Needle passes(times)	2–5	11(5.1)	0.58	0.217–1.550	0.272
	6-	7(8.4)	1		
Sex	Male	2(1.3)	1		
	Female	16(11.0)	9.488	2.142–42.093	0.0004
HBV infection	negative	11(4.4)	1		
	positive	7(13.5)	3.352	1.233–9.108	0.013
Renal disease	negative	17(6.1)	1		
	positive	1(4.5)	0.731	0.093–5.766	0.765
Diabetes	negative	18(6.3)			
	positive	0(0)			1.00

Data are expressed as number and %

*Abbreviations*: *Group U* nerve block with ultrasound guidance, *Group N* nerve block with nerve stimulation-guidance, *group M* nerve block with combined guidance

## Discussion

There were three main findings in the current study. First, the use of ultrasound did not improve the quality of deep nerve block. Second, the use of ultrasound increased the incidence of LAST. Third, the use of ultrasound, HBV infection and the female sex may be risk factors of LAST.

In the present study, we found that LPBs and SNBs with US were not superior to those with NS in terms of the onset or restoration time. Spencer S. Liu et al. [19] found that 8 of 10 RCTs reported that the use of ultrasound would shorten the onset time of lower extremity blocks, 2 of 10 reported no difference, and no RCTs reported slower onset with ultrasound. However, most of the RCTs were about the femoral and peroneal nerves. Recently, Arnuntasupakul et al. [12] reported that ultrasound with nerve stimulation guidance for LPBs resulted in a shorter total anesthesia and onset times than US alone. Due to the size and location of the lumbar plexus and sciatic nerves, LPBs and SNBs are advanced regional anesthesia techniques. Different induction pathways for the nerve block may result in different outcomes. Furthermore, due to comorbidities, some of the enrolled patients may have already had minor pathological changes in the targeted nerves, which may have affected the onset and restoration times.

The incidence of LAST was 6%,which is much higher than previously reported [1]. LAST can occur as a result of the patient’s risk factors and current medications, inadvertent injection of LAs directly into the vascular system, exceeding the maximum LA dose, or immediate LA absorption upon injection into an extremely vascularized area [18]. It has been widely reported that US is safer than NS because US can provide direct visualization of the target nerve, surrounding tissues, and LA spread [9, 20]. However, in our study, approximately two-thirds of the patients who experienced LAST were in group U. There are two main reasons for this finding. First, a fair number of patients in our study were infected with HBV or had a renal diseases and thus may be more susceptible to LAST [21]. Second, the lumbar plexus and sciatic nerves are difficult to visualize due to their depth. To obtain a better view of tissues near the nerve, the block needle and the injected LAs, an ultrasonic probe must be applied with some pressure near the injection site. This pressure slows the blood flow in the deep, small vessels, and it is difficult to examine the deep, small vessels using Doppler ultrasonography, especially with slow flow [22]. The continuous pressure caused by the ultrasonic probe makes deep, small vessels “invisible” and thus increased the difficulty of avoiding injecting LAs into extremely vascularized areas, resulting in LAST. The nerve stimulator has advantages over US in terms of determining the relative positions of the needle tip and nerves. When the needle tip is near an extremely vascularized area, the electric current decreases, resulting in failure to induce muscle twitching. In group M, after ultrasound guidance, the rate of failure to induce twitching in the quadriceps and gastrocnemius distribution was 10 and 12%, respectively. Under these circumstances, the distance between the tip and the targeted nerve needed to be adjusted. Therefore, the likelihood of injecting LAs into extremely vascularized areas was less than that with US alone.

The patient’s weight, comorbidities, use of other medications, genetics, allergies, and other physiological limitations also affecting the incidence of LAST [23]. Factors affected systemic LA absorption, the peak plasma LA concentration and the time to reach that peak are all related to LAST. Bupivacaine and ropivacaine are degraded in the liver by α1-acid glycoprotein (AAG) [21]. Patients with liver diseases would have a decreased rate of LAs clearance due to a reduced AAG concentration, which may increase the incidence of LAST. However, even in patients with advanced liver dysfunction, the synthesis of AAG is still maintained [21, 24]. In patients with hepatic dysfunction, single-dose blocks can usually be performed safely with a normal dose of LAs [25]. This finding indicates that the decreased clearance of LAs caused by isolated hepatic dysfunction is not the main reason for LAST in this study. However, as shown in Tab 4, patients infected with HBV had a higher risk of LAST in this study. Patients who are infected with HBV may develop chronic liver disease. Patients with chronic liver disease usually have vascular dysfunction, especially angiogenesis, microvascular derangements and microcirculatory dysfunction [26, 27]. Cirrhosis causes numerous microscopic vessel aberrations, and these vessels may become entangled with each other, resulting in sharp bends, anomalous branching patterns, abnormal branching angles and tortuosity [28]. McAvoy et al. [29] demonstrated that patients with cirrhosis had selective regional increases in blood flow in the splanchnic and hepatic circulations but diminished flow in the peripheral limbs. Neovascularization and slower blood flow make it easier to inject LAs into extremely vascularized areas, especially for the use of ultrasound, resulting in an increased incidence of LAST. Vascular endothelial growth factor (VEGF) and bone morphogenetic protein 9 (BMP-9) have been widely reported to promote angiogenesis [30]. Higher BMP-9 levels in human serum are accompanied by advanced stages of liver fibrosis, while BMP-9 overexpression accelerated liver fibrosis and BMP-9 knockdown attenuated the liver fibrosis in a mouse model [31]. The plasma VEGF level was elevated in patients with cirrhosis, especially in those with spider angiomas [27]. Higher serum levels of BMP-9 and VEGF in patients with HBV indicated more advanced stages of liver dysfunction and increased new blood vessel formation. However, further efforts are needed to determine the relationship of VEGF and BMP-9 with HBV infection. VEGF and BMP-9 may be promising prognostic indicators for the incidence of LAST after deep nerve block in patients with HBV.

Herein, women were more likely to experience LAST, which is in consistent with the latest regional anesthesia and pain medicine practice advisory on LAST [1]. Some enrolled patients who experienced LAST were HBV carriers. The increased risk of LAST in females may be related to HBV infection. Under physiological conditions, the estrogen/estrogen receptor α (ER/ER α) axis has a protective effect against HBV-associated liver damage, and postmenopausal hormone replacement therapy results in a lower risk of hepatocellular carcinoma in HBV positive women [32]. In a female cirrhosis rat model the mRNA expression of ER α was lower in that of a sham rats and the ability of 17β-estradiol to alleviate relevant complications was diminished [33]. This finding indicates that female HBV carriers might have a lower level of ER/ER α, which made them more susceptible to LAST. Further efforts are needed to investigate the underlying mechanism.

There are a number of limitations to this study. First, it was not possible to blind the anesthesiologist performing the nerve block, and we could not exclude the potential influence of a performance bias in this study. Second, although we made efforts to maintain blinding among the investigators, patients, and statistician, it may be partial blinding due to the muscle contractions elicited by nerve stimulation, counting the number of needle redirections and so on. We attempted to minimize this bias by only involving staff anesthesiologists experienced in peripheral nerve blockades using both guidance modalities. Third, there is some limitation related to the techniques and equipment used in this single-center study, so the results cannot be generalized to other techniques or peripheral nerve block locations. The degree of advantages and disadvantages provided by ultrasound guided deep nerve block, especially in HBV carriers, is likely to vary by the nerve block site as well. We only demonstrated some interesting phenomenon and did not determine the underlying mechanisms in the present single-center study. A multicenter study and more detailed experiments are needed to verify our results and reveal the mechanisms.

## Conclusions

Our results suggest that ultrasound guidance, HBV infection and the female sex are risk factors of LAST in LPBs and SNBs. For patients infected with HBV or female patients undergoing LPBs and SNBs, combined ultrasound and nerve stimulation guidance should be used to improve the safety. The probable mechanisms are as follows:1) angiogenesis and slower blood flow in deeply located small vessels; and 2) the use of nerve stimulation to avoid injecting LAs into extremely vascularized areas.

## Abbreviations

BMP-9: Bone Morphogenetic Protein 9
LAs: Local Anesthetics
LAST: Local Anesthetics Systemic Toxicity
LPBs: Lumbar Plexus Blocks
NS: Nerve Stimulator guidance
SNBs: Sciatic Nerve Blocks
US: Ultrasound guidance
VEGF: Vascular Endothelial Growth Factor
